# Supplementary material for: Secreted EMC10 is upregulated in human obesity and its neutralizing antibody prevents diet-induced obesity in mice
Source: Nat Commun. 2022 Nov 28;13:7323. doi: 10.1038/s41467-022-34259-9 (PMC9705309; doi:10.1038/s41467-022-34259-9)
Supplement: Supplementary file 1 — Supplementary Information [file 41467_2022_34259_MOESM1_ESM.pdf]

Supplementary Materials for

Secreted EMC10 is upregulated in human obesity and its neutralizing antibody prevents diet-induced obesity in mice

Xuanchun Wang[1#*], Yanliang Li[1,2,3#], Guifen Qiang[2,4#], Kaihua Wang[5,6#], Jiarong Dai[1], Maximilian McCann[2], Marcos D Munoz[2], Victoria Gil[2], Yifei Yu[1], Shengxian Li[2,7], Zhihong Yang[8,9], Shanshan Xu[2], Jose Cordoba-Charcon[10], Dario F De Jesus[8], Bei Sun[11], Kuangyang Chen[1], Yahao Wang[1], Xiaoxia Liu[1], Qing Miao[1], Linuo Zhou[1], Renming Hu[1], Qiang Ding[12], Rohit N Kulkarni[8], Daming Gao[3,13], Matthias Blüher[14], Chong Wee Liew[2*]

[1]Department of Endocrinology, Huashan Hospital, Fudan University, Shanghai, China

[2]Department of Physiology & Biophysics, University of Illinois at Chicago, Chicago, IL, USA

[3]Department of Ophthalmology and Visual Sciences, University of Illinois at Chicago, Chicago, IL, USA (Current affiliation)

[4]State Key Laboratory of Bioactive Substances and Functions of Natural Medicines, Institute of Materia Medica, Chinese Academy of Medical Sciences and Peking Union Medical College, Beijing, China (Current affiliation)

[5]State Key Laboratory of Cell Biology, Shanghai Institute of Biochemistry and Cell Biology, CAS Center for Excellence in Molecular Cell Science, Chinese Academy of Sciences, Shanghai, China

[6]University of Chinese Academy of Sciences, Beijing, China

[7]Department of Endocrinology and Metabolism, Renji Hospital, School of Medicine, Shanghai Jiao Tong University, Shanghai, China

[8]Research Division, Joslin Diabetes Center, Harvard Medical School, Boston, MA, USA

[9]Department of Transplant Surgery, Mass General Hospital, Harvard Medical School, Boston, MA, USA (Current affiliation)

[10]Department of Medicine, Section of Endocrinology, Diabetes and Metabolism, University of Illinois at Chicago, Chicago, [11]NHC Key Laboratory of Hormones and Development, Tianjin Key Laboratory of Metabolic Diseases, Chu Hsien-I Memorial Hospital & Tianjin Institute of Endocrinology, Tianjin Medical University, Tianjin, China

[12]Department of Urology, Huashan Hospital, Fudan University, Shanghai, China

[13]Key Laboratory of Systems Health Science of Zhejiang Province, School of Life Science, Hangzhou Institute for Advanced Study, University of Chinese Academy of Sciences, Hangzhou, China

[14]Department of Medicine, University of Leipzig, Leipzig, Germany

# Equal contribution

* Co-corresponding author

Corresponding author:

Chong Wee Liew PhD

Department of Physiology & Biophysics

College of Medicine

University of Illinois at Chicago

835 S Wolcott Ave, M/C 901, MSB, E-202, Chicago, IL 60612

Telephone: 1-312-413-1086; Fax: 1-312-996-1414
E-mail: cwliew@uic.edu

Xuanchun Wang, M.D., Ph.D.

Institute of Endocrinology and Diabetes,

Department of Endocrinology and Metabolism,

Huashan Hospital, Fudan University,

12 Wulumuqi Zhong Road, Shanghai 200040, China

Tel: +86-21-52888286 Cell Phone: +86-13818922969

Email: wangxch@fudan.edu.cn

Keywords: EMC10, obesity, secreted factor, thermogenesis, brown adipose tissue, neutralizing antibody

This PDF file includes:

Supplementary Figures 1 to 9

Supplementary Tables 1 to 4

Supplementary Figure 1

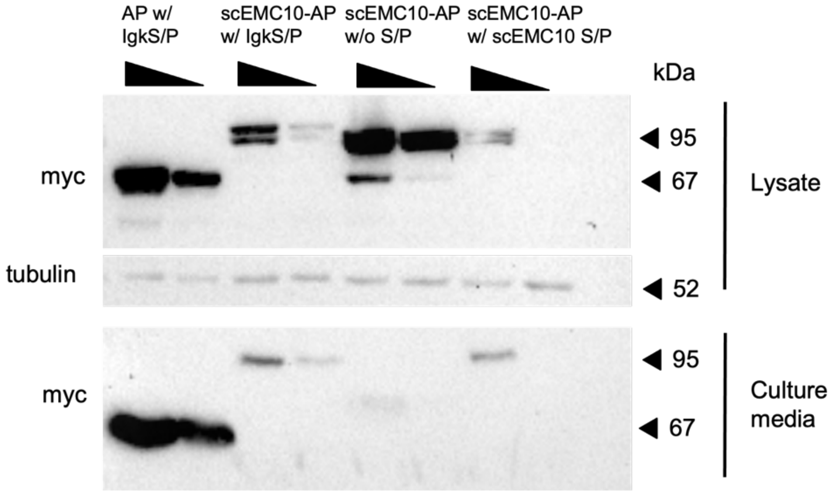

Supplementary Figure 1. *In vitro* confirmation of scEMC10 as a secreted protein

Western blotting for cell lysate or culture media from 293T cells 48h after transfection with myc-tagged alkaline phosphatase or scEMC10-AP with or without Igk signal peptide or scEMC10 endogenous signal peptide plasmid constructs. AP, alkaline phosphatase; S/P, signal peptide; w/, with; w/o, without. The experiment was repeated 2 times independently with similar results

Supplementary Figure 2

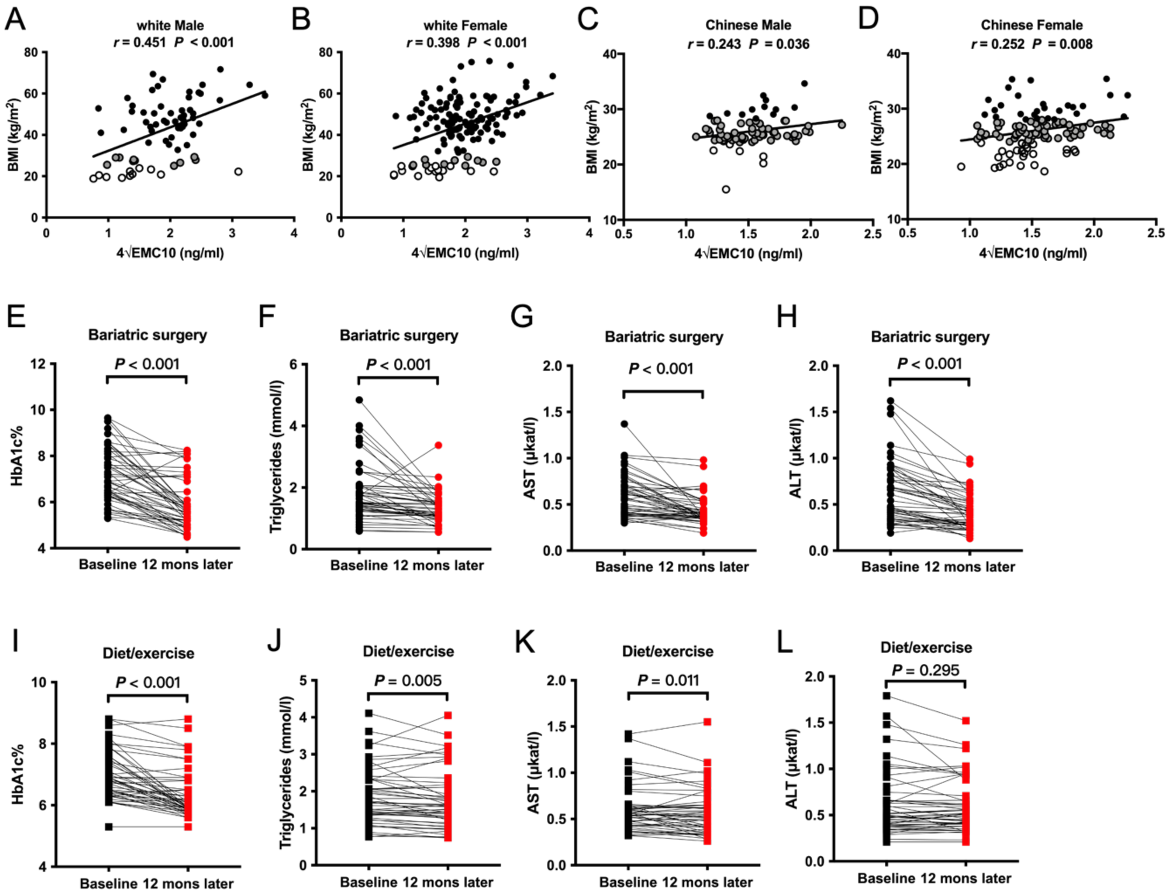

Supplementary Figure 2. Association of serum EMC10 levels with BMI in humans and changes of metabolic parameters before and after weight loss in patients with obesity

(A-D) Associations of serum EMC10 levels with BMI in male or female white or Chinese Han cohort including subjects with leanness, overweight and obesity. The correlation analyses were performed using Pearson's bivariate correlation. Changes of HbA1c, serum triglycerides, AST, and ALT before and 12 months after bariatric surgery **(E-H)** or diet/exercise **(I-L)** in subjects of a white weight-loss cohort. Comparisons between before and after were performed using Student's paired t-test. For all analyses, two-side P values < 0.05 were considered statistically significant.

Supplementary Figure 3

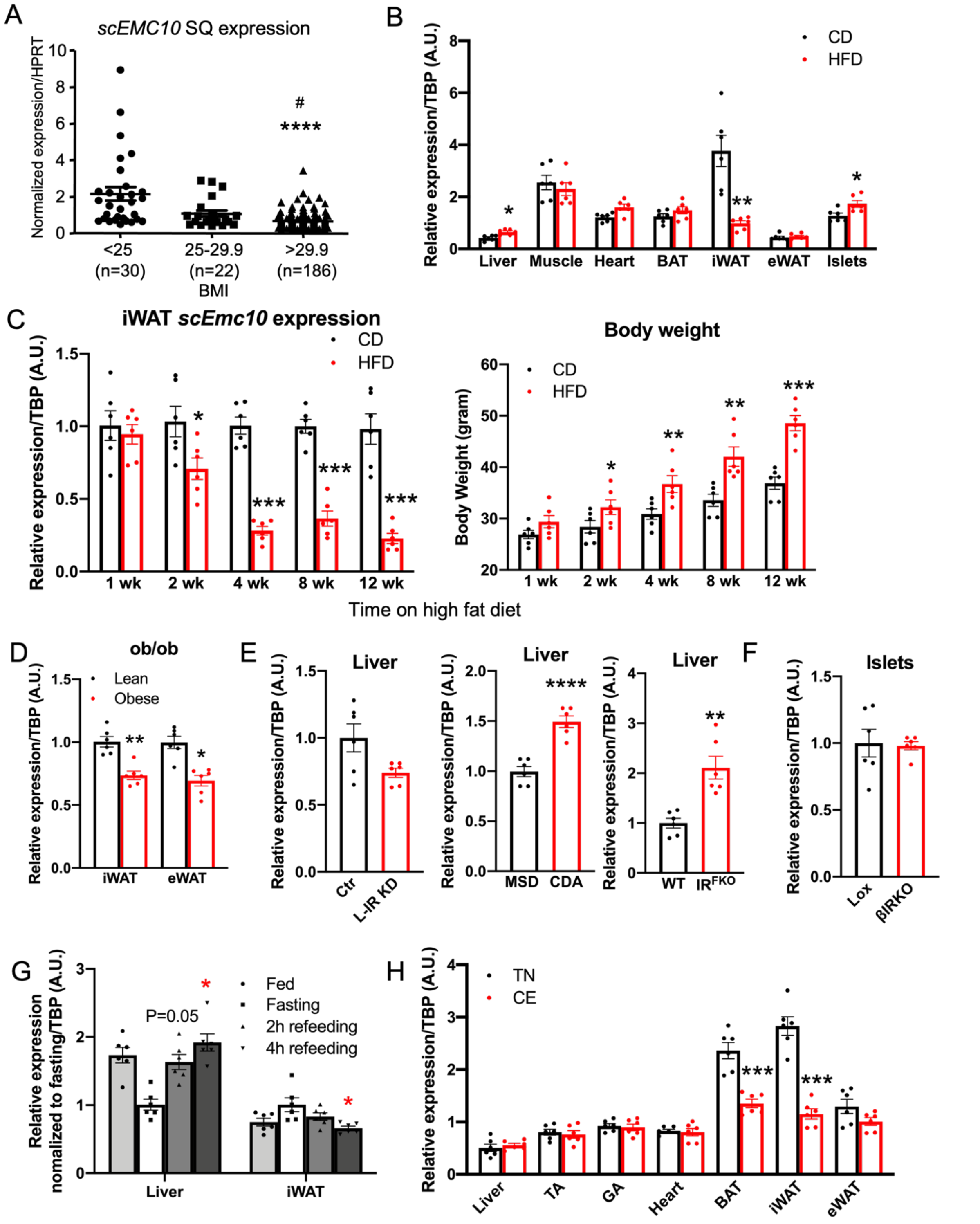

Supplementary Figure 3. Regulation of *scEMC10* gene expression in humans and mouse models

(A) Human *scEMC10* mRNA in subcutaneous (SQ) fat from patients with leanness, overweight and obesity. (n number indicated). All data are presented as mean +/- SEM. ****, p<0.0001 (vs <25); #, p<0.05 (vs 25-29.9). **(B)** *scEmc10* mRNA in liver, muscle, heart, brown adipose tissue (BAT), subcutaneous (inguinal, iWAT), visceral (epididymal, eWAT) white adipose tissue and pancreatic islets from chow diet (CD) or high fat diet (HFD) fed C57BL/6 mice (n=6 per group). **(C)** iWAT (inguinal white adipose tissue) *scEmc10* mRNA

levels and body weights of C57BL/6 mice on HFD (n=6 per group). **(D)** *scEmc10* mRNA in subcutaneous (inguinal, iWAT), and visceral (epididymal, eWAT) white adipose tissue from lean and obese ob/ob mice (n=6 per group). **(E)** *scEmc10* mRNA in liver from WT and IR^{FKO} (adipose tissue-specific insulin receptor KO) mice, or C57BL/6 mice fed with control (MSD) or choline-deficient and methionine-restricted (0.1%), high-fat diet (60% Kcal from fat) (CDA), or IR^{flox} mice 7-days after injection with AAV-GFP (Ctr) or AAV-Cre (acute liver insulin receptor knockdown model, L-IR KD) (n=6 per group). **(F)** *scEmc10* mRNA in pancreatic islets from control (Lox) and beta-cell specific insulin receptor KO (βIRKO) mice (n=3). **(G)** *scEmc10* mRNA in liver or subcutaneous (inguinal, iWAT) white adipose tissue from C57BL/6 mice in the fed, 24h fasting, 2h or 4h refeeding states (n=6 per group). **(H)** *scEmc10* mRNA in liver, tibialis anterior (TA) and gastrocnemius (GA) muscle, heart, brown adipose tissue (BAT), subcutaneous (inguinal, iWAT) and visceral (epididymal, eWAT) white adipose tissue from thermoneutral (TN) or cold exposure (CE) treated C57BL/6 mice (n=6 per group). All data are presented as mean +/- SEM. Statistical significance was assessed by two-sided Student's t test and significant differences were indicated with p values. Source data are provided in the Source Data file.

Supplementary Figure 4

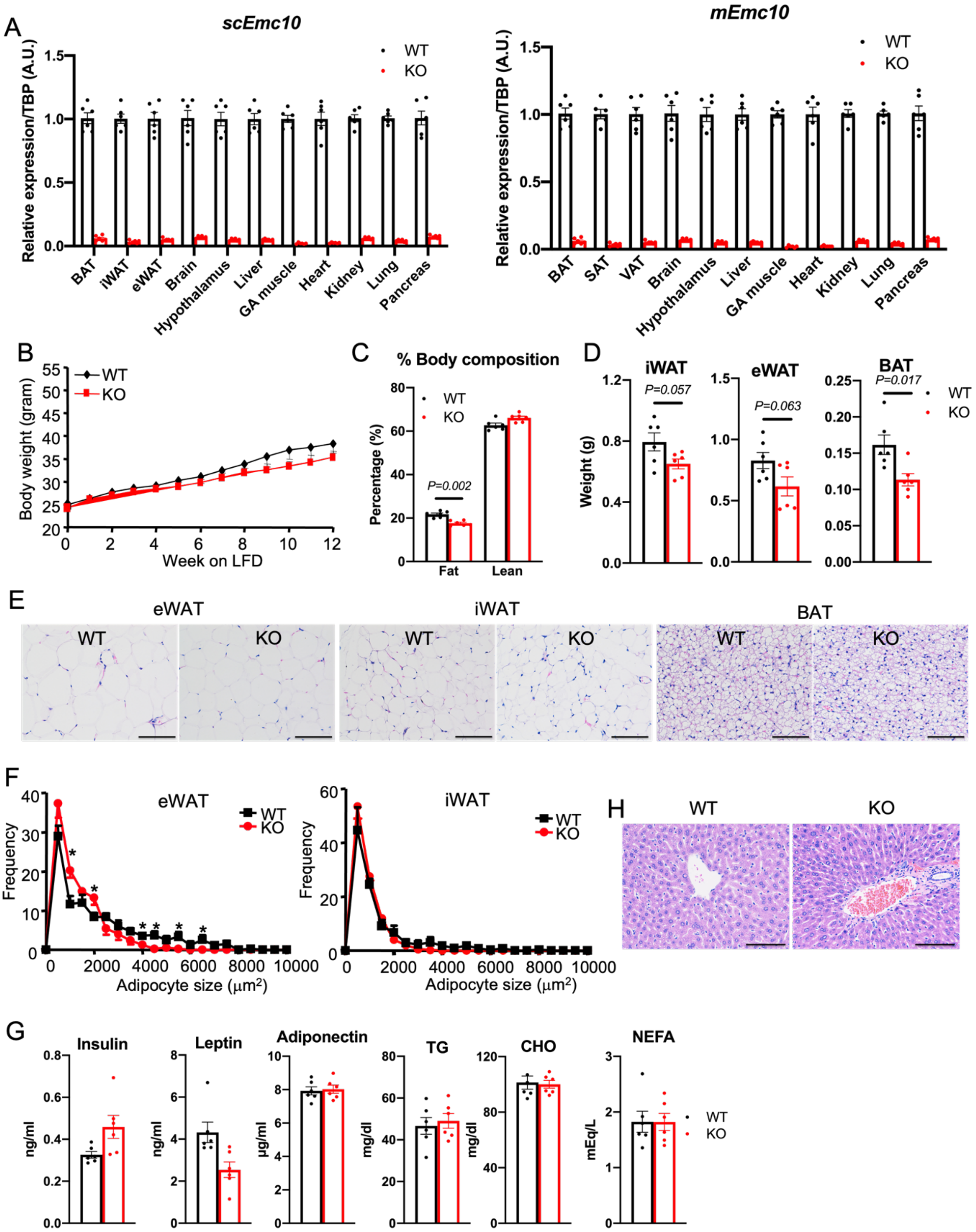

Supplementary Figure 4. Phenotypes of *Emc10* knockout mice fed with low fat diet (LFD)

(A) *scEmc10* and *mEmc10* mRNA in brown (BAT), subcutaneous (SAT) and visceral (VAT) adipose tissue, brain, hypothalamus, liver, gastrocnemius (GA) muscle, heart, kidney, lung and pancreas tissue dissected from male WT (open) or KO (black) CD-fed mice (n=6 per group). **(B)** Body weights of male (WT, black diamond), and KO (red square) on C57BL/6 background on LFD (n=6 per group). Percentage of body composition **(C)**, and weights of subcutaneous (inguinal, iWAT), visceral (epididymal, eWAT) and brown

(BAT) adipose tissues **(D)** from male WT (open), and KO (red) mice fed with 12-wks of LFD (n=6 per group). **(E)** Representative images of hematoxylin and eosin (H&E)-stained sections of eWAT, iWAT, and BAT from male WT and KO mice fed LFD. Scale bar, 100um. **(F)** Quantification of eWAT and iWAT adipocyte size from male WT (black square) and KO (red circle) mice fed LFD. (n=8 per group). **(G)** Plasma insulin, leptin, adiponectin, triglyceride (TG), cholesterol (CHO), and non-esterified fatty acid (NEFA) in male WT (open), and KO (red) mice fed with 12-wks of LFD in the fed state (n=6 per group). **(H)** Representative images of H&E-stained sections of livers from male WT and KO mice fed with 12-wks of LFD. Scale bar, 100um. All data are presented as mean +/- SEM. Statistical significance was assessed by two-sided Student's t test (A, C, D, F, G) and significant differences were indicated with p values. *, $p<0.05$. Source data are provided in the Source Data file.

Supplementary Figure 5

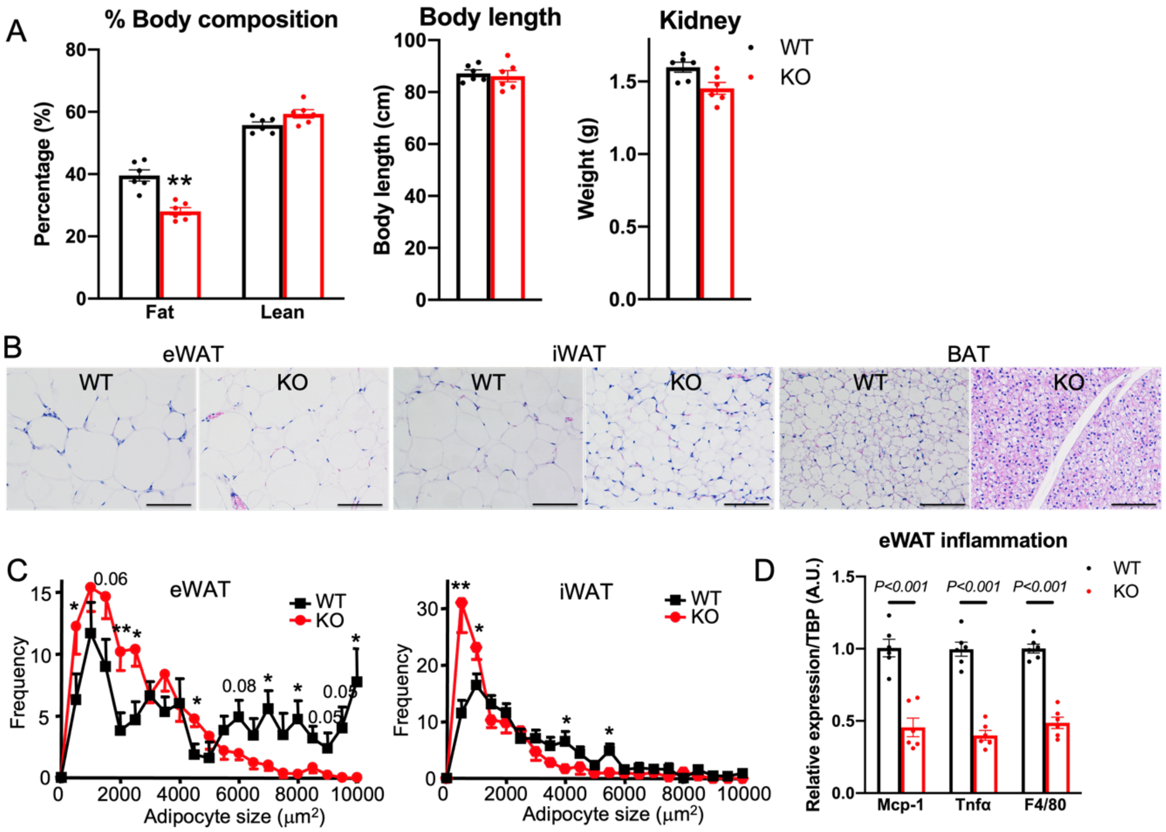

Supplementary Figure 5. Phenotypes of *Emc10* knockout mice fed with high fat diet (HFD)

(A) Percentage of body composition, body length and kidney weight of male WT (open), and KO (red) mice fed with 12-wks of HFD (n=6 per group). **(B)** Representative images of hematoxylin and eosin (H&E)-stained sections of visceral (epididymal, eWAT), subcutaneous (inguinal, iWAT), and brown (BAT) adipose tissue from male WT and KO mice fed HFD. Scale bar, 100um. **(C)** Quantification of eWAT and iWAT adipocyte size from male WT (black square) and KO (red circle) mice fed HFD. (n=16 & 12 per group). **(D)** *Mcp-1*, *Tnfa*, and *F4/80* mRNA in eWAT from male WT (open), and KO (red) mice fed with 12-wks of HFD (n=6-8 per group). All data are presented as mean +/- SEM. Statistical significance was assessed by two-sided Student's t test (A, C & D)and significant differences were indicated with p values. *, p<0.05; **, p<0.01. Source data are provided in the Source Data file.

Supplementary Figure 6

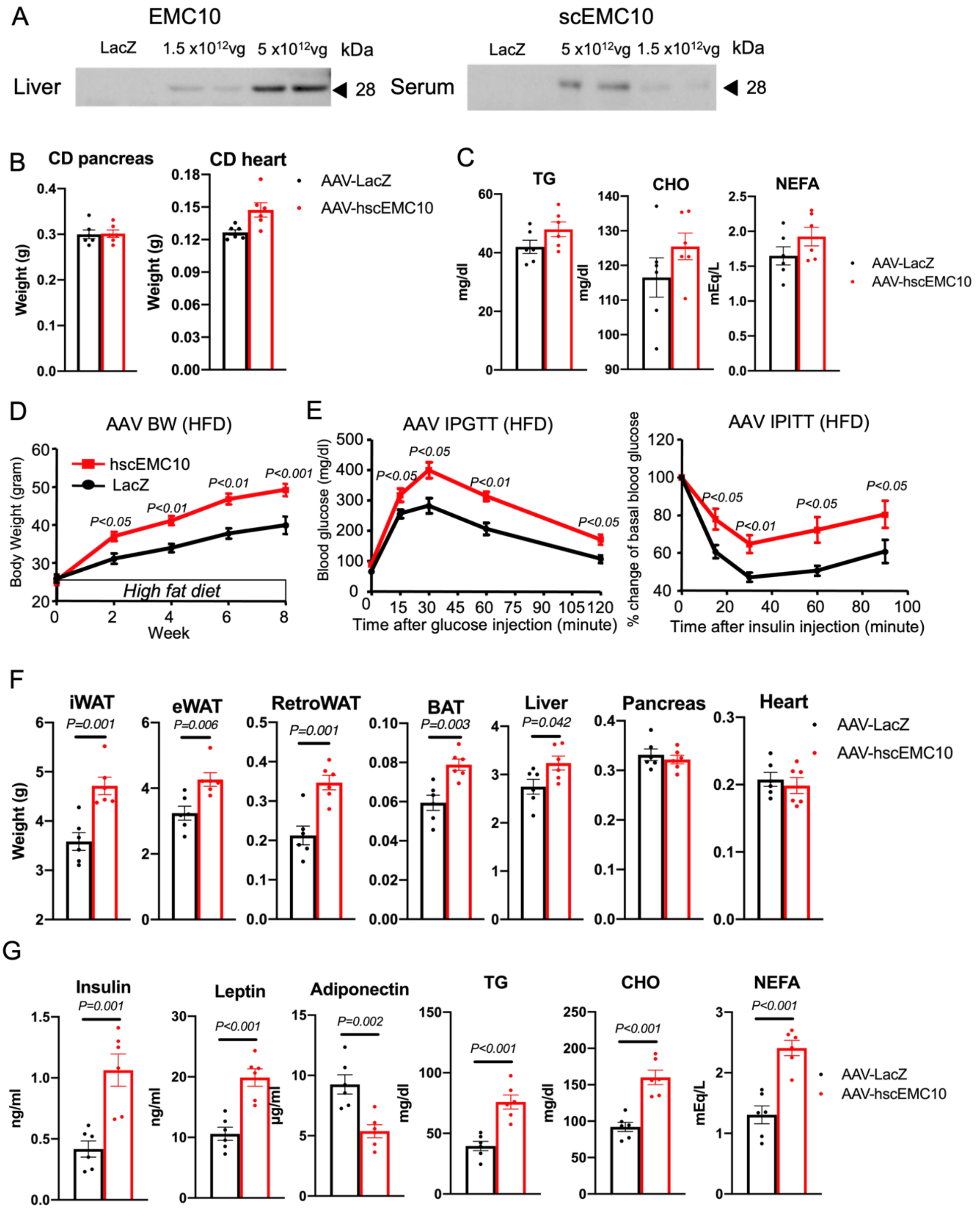

Supplementary Figure 6. Phenotypes of *hscEMC10* overexpressed mice

(A) Representative western blotting for EMC10 protein in liver and serum from C57BL/6 mice after injected with AAV-LacZ or AAV-*hscEMC10*. **(B)** Pancreas and heart weights of C57BL/6 mice fed with 18-wks of chow diet (CD) after injection with AAV-LacZ or AAV-*hscEMC10* (n=6 per group). **(C)** Plasma triglyceride (TG), cholesterol (CHO) and non-esterified fatty acid (NEFA) of C57BL/6 mice fed with 18-wks of CD after injection with AAV-LacZ or AAV-*hscEMC10* (n=6 per group). **(D)** Body weights of male C57BL/6 mice expressing LacZ control or *hscEMC10* via tail-vein AAV transduction after 8-wks of HFD (n=6 per group). **(E)** Glucose tolerance (left) and insulin tolerance (right) of male C57BL/6 mice expressing LacZ control or

hscEMC10 via tail-vein AAV transduction after 8-wks of HFD (n=6 per group). **(F)** Weights of subcutaneous (inguinal, iWAT), visceral (epididymal, eWAT), retroperitoneal (retroWAT) and brown (BAT) adipose tissue, and liver, pancreas, and heart of male C57BL/6 mice expressing LacZ control or *hscEMC10* via tail-vein AAV transduction after 8-wks of HFD (n=6 per group). **(G)** Plasma insulin, leptin, adiponectin, TG, CHO, and NEFA of male C57BL/6 mice in the fed state expressing LacZ control or *hscEMC10* via tail-vein AAV transduction after 8-wks of HFD (n=6 per group). All data are presented as mean +/- SEM.. Statistical significance was assessed by two-sided Student's t test (B--G) and significant differences were indicated with p values. Source data are provided in the Source Data file.

Supplementary Figure 7

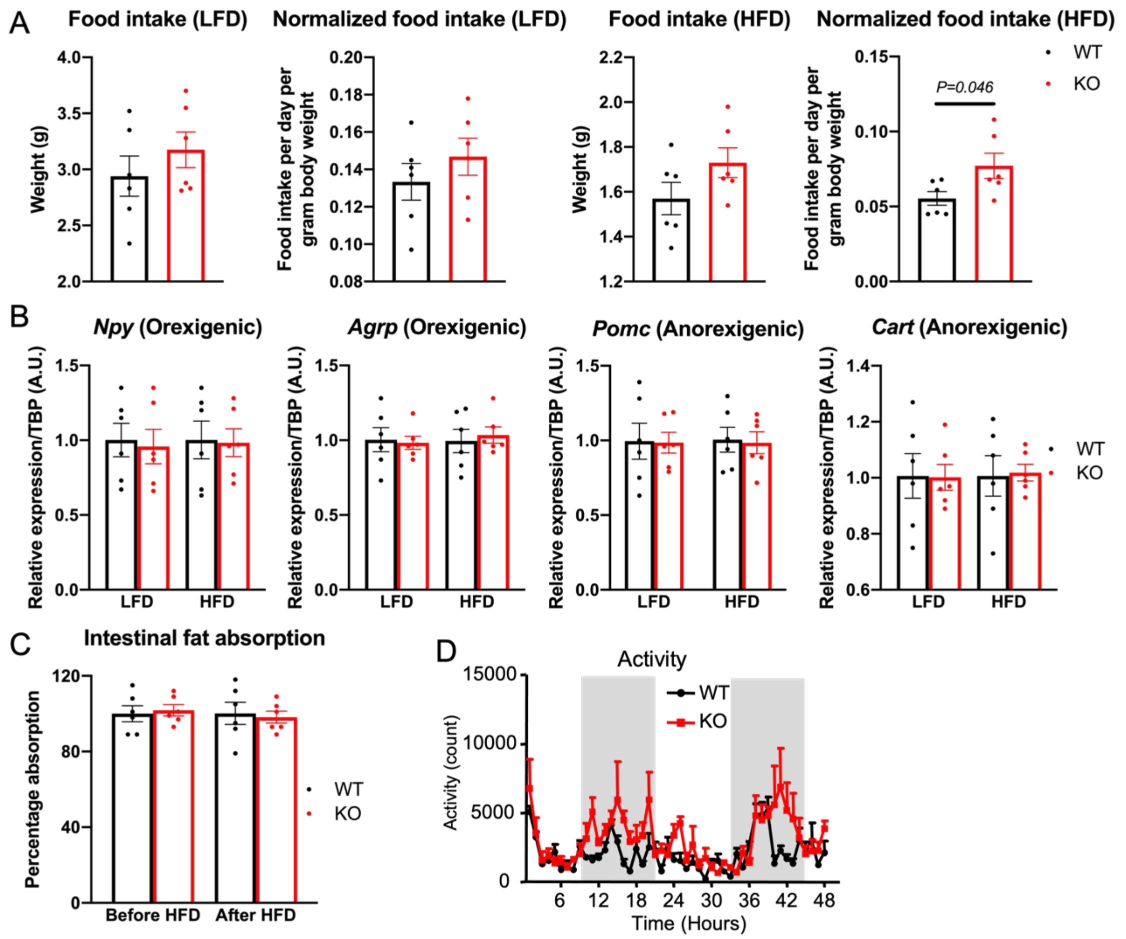

Supplementary Figure 7. Energy metabolism of wildtype (WT) and *Emc10* knockout (KO) mice

(A) Food intake and body weight-normalized food intake of male WT (open), and KO (red) mice fed with 12-wks of LFD or HFD (n=6 per group). **(B)** Orexigenic and anorexigenic peptide mRNA expression in hypothalamus from male WT (open), and KO (red) mice fed with 12-wks of LFD or HFD (n=6 per group). **(C)** Intestinal fat absorption of male WT (open), and KO (red) mice before or fed with 12-wks of HFD (n=6 per group). **(D)** Physical activity of male WT (circle, black), and KO (square, red) mice fed with 12-wks of HFD (n=6 per group). All data are presented as mean +/- SEM. Statistical significance was assessed by two-sided Student's t test and significant differences were indicated with p values. Source data are provided in the Source Data file.

Supplementary Figure 8

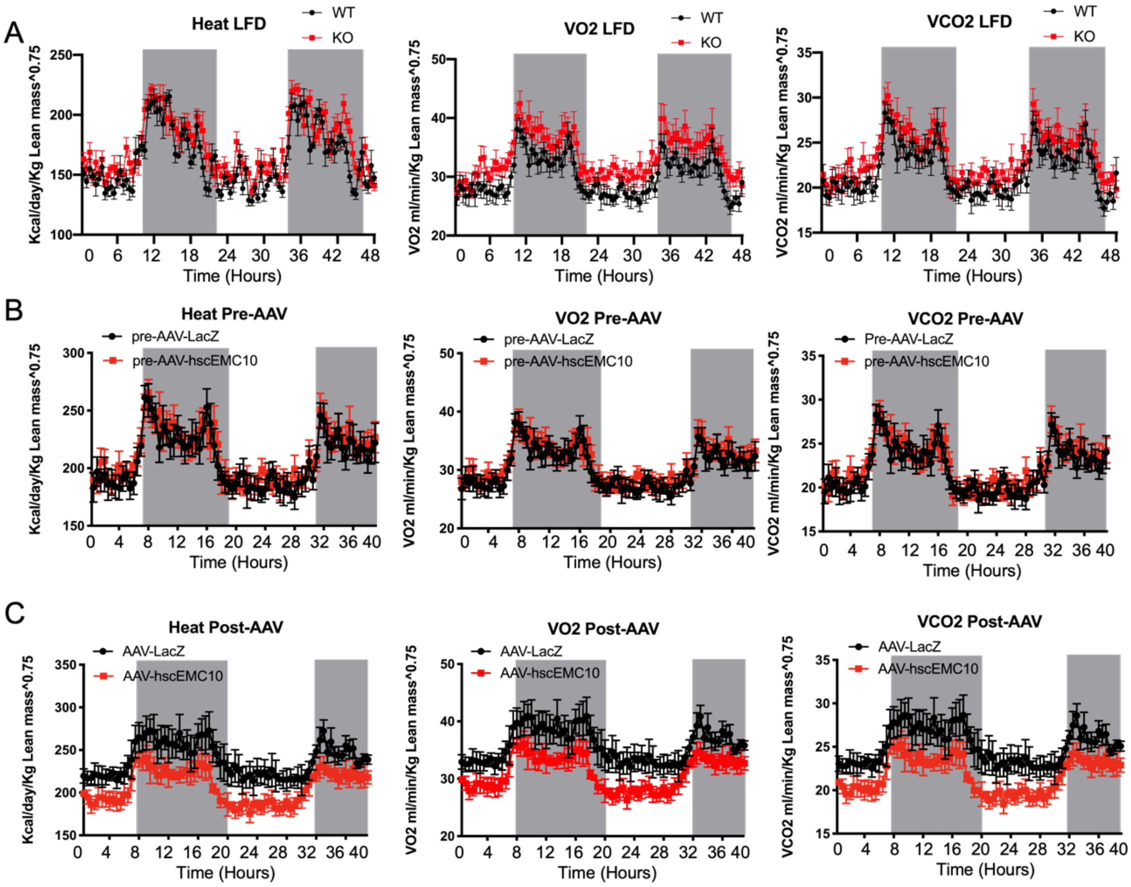

Supplementary Figure 8. Energy expenditure in *Emc10* KO or scEMC10 overexpressed mice

(A) Heat production (left), oxygen consumption (VO2) (middle) and carbon dioxide production (VCO2) (right) were analyzed by indirect calorimetry for 48h in WT (black circle) or KO (red square) mice under LFD (n=8 per group). **(B)** Heat production (left), oxygen consumption (VO2) (middle) and carbon dioxide production (VCO2) (right) were analyzed by indirect calorimetry for 40h in B6 mice before AAV injection (n=6 per group). **(C)** 10 days after either AAV-Laz control (black circle) or AAV-hscEMC10 (red square) injection, heat production (left), oxygen consumption (VO2) (middle) and carbon dioxide production (VCO2) (right) were analyzed by indirect calorimetry for 40h (n=6 per group). All data are presented as mean +/- SEM.

Supplementary Figure 9

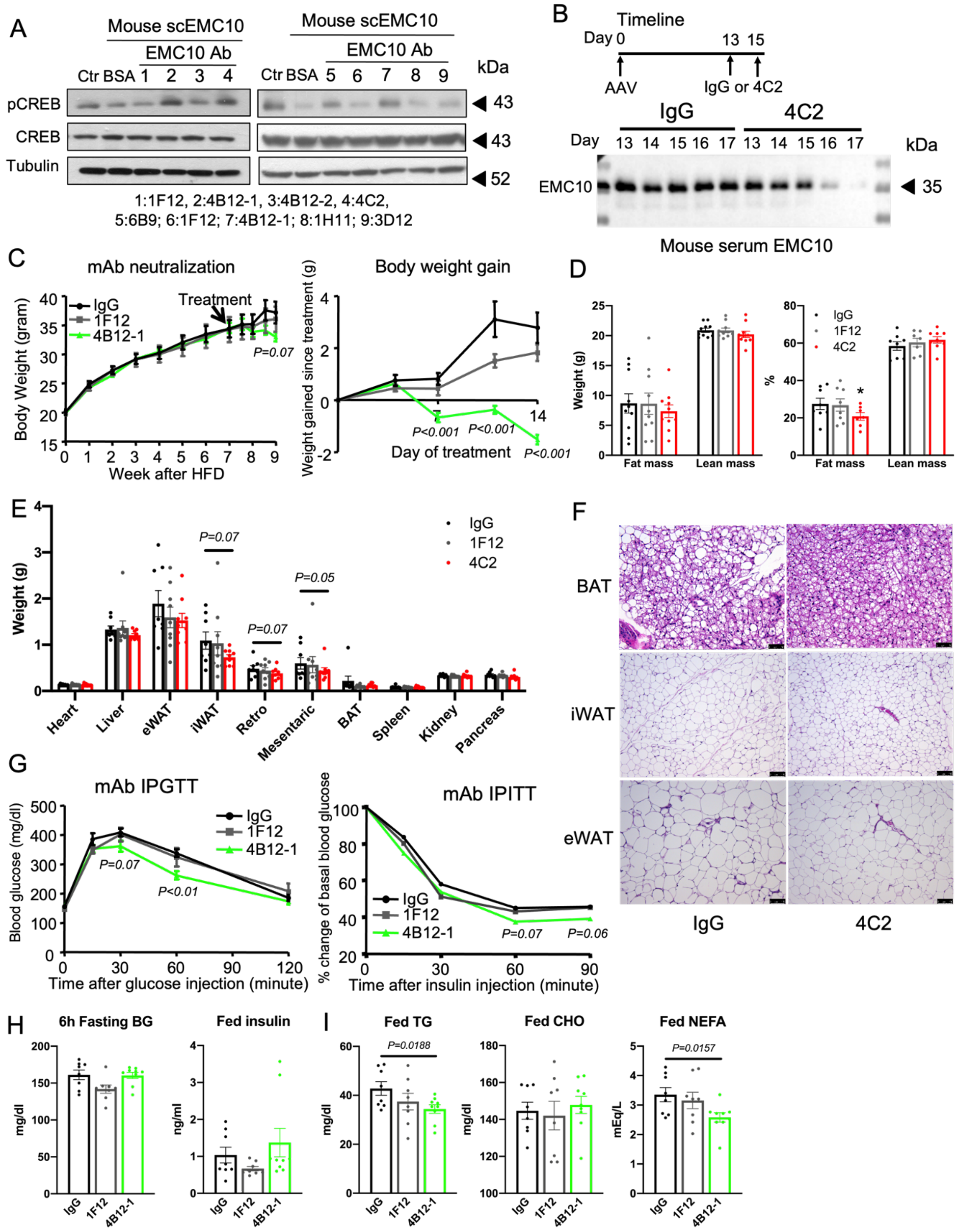

Supplementary Figure 9. Phenotypes of mice treated with scEMC10 monoclonal antibodies

(A) Western blotting for phospho (Ser 133)-CREB, total CREB and tubulin in HeLa cells treated with media containing 1 μg/ml mouse scEMC10 protein and 1 μg/ml individual mouse anti-human scEMC10 monoclonal antibody or BSA as indicated for 6 h, Ctr: Control medium without mouse scEMC10 protein nor antibodies. Experiment was repeated 3 times. **(B)** Western blotting for scEMC10 in serum from *scEmc10* over-expressor mice before and after treatment with either IgG or 4C2 antibodies. **(C)** Body weight (left)

and body weight gain (right) of C57BL/6J male mice fed with HFD before or after IP injected with 3 mg/kg BW antibody as indicated twice a week. (IgG, black circle. mAb-1F12, grey square. mAb-4B12-1, green triangle) (n = 9 per group). **(D)** Body composition of mice IP injected with IgG (black), 1F12 (grey) or 4C2 (red) antibodies determined by DEXA (n=8, 9 & 9 per group). **(E)** Tissue {heart, liver, eWAT, iWAT, retroperitoneal (Retro) and mesenteric adipose tissue, BAT, spleen, kidney, pancreas} weight from male mice treated with IgG (black), 1F12 (grey) or 4C2 (red) antibodies (n=8-10 per group). **(F)** Representative images of H&E-stained sections of BAT, iWAT and eWAT from mice treated with IgG or 4C2 antibodies. Scale bar, 50um. **(G)** Glucose tolerance (left) and insulin tolerance (right) of mice treated with IgG (black circle), 1F12 (grey square) or 4B12-1 (green triangle) antibodies (n=9 per group). **(H)** Plasma glucose after 6h fasting and fed insulin in male mice treated with IgG (black), 1F12 (grey) or 4B12-1 (green) antibodies (n=8-10 per group). **(I)** Fed plasma non-esterified fatty acid (NEFA), triglyceride (TG), and cholesterol (CHO) in mice treated with IgG (black), 1F12 (grey) and 4B12-1 (green) antibodies (n=9 per group). All data are presented as mean +/- SEM. Statistical significance was assessed by two-sided Student's t test (G-I) and significant differences were indicated with p values. Source data are provided in the Source Data file.

Supplementary Table 1. Anthropometric parameters and clinical characteristics of white participants (n=240).

	BMI≤24.9<br>N=30	25≤BMI≤29.9<br>N=22	BMI≥30<br>N=188	*P*-value
Sex, female/male (n)	18/12	12/10	134/54	0.156
Age (years)	61.53±20.10	65.08±14.34	47.93±11.32*, #	<0.001
BMI (kg/m^2)	22.10±1.95	27.35±1.50*	49.84±9.30*, #	<0.001
FPG (mmol/l)^{φ}	5.31(4.77,5.90)	5.29(4.92,6.28)	5.72(5.10,7.53)*	0.002
Haemoglobin A1c (%)^{φ}	5.10(4.90,5.40)	5.50(5.20,5.85)	6.05(5.40,6.80)	<0.001
Diabetes n (%)	2 (6.7)	3 (13.6)	98 (52.1)*, #	<0.001
Triglycerides (mmol/l)^{φ}	0.94(0.83,1.07)	1.01(0.81,1.41)	1.67(1.15,2.34)*, #	<0.001
TC (mmol/l)	4.73±0.69	4.90±1.29	5.02±1.08	0.703
ALT (U/L)^{φ}	22.80(13.20,31.80)	23.4(20.4,33.00)	33.00(23.40,45.60)*, #	<0.001
AST (U/L)^{φ}	25.20(19.80,27.60)	24.90(19.80,33.60)	28.80(21.00,37.20)	0.050
Creatinine (µmol/l)	67.00(58.00,73.00)	68.00(61.00,79.50)	70.72(61.88,83.00)	0.145

Data indicate means ± SD or medians (interquartile range). One-way ANOVAs with Fisher's *LSD post hoc* test were used to compare the differences among the three groups. *, $P < 0.05$ compared to lean; #, $P < 0.05$ for difference between the obese versus overweight groups. Abbreviations: BMI, body mass index; FPG, fasting plasma glucose; AST, aspartate transaminase; ALT, alanine aminotransferase; TC, total cholesterol; ^{φ}Log transformed before analysis.

Supplementary Table 2. Anthropometric parameters and clinical characteristics of Chinese Han participants (n=186).

	BMI≤24.9<br>N=32	25≤BMI≤29.9<br>N=115	BMI≥30<br>N=39	*P*-value
Sex, female/male (n)	26/6	58/57	27/12	0.003
Age (years)	50.41±5.95	51.25±6.83	52.64±7.85	0.376
BMI (kg/m2)	21.50±1.85	25.82±1.12*	30.51±2.12*, #	<0.001
FPG (mmol/l)φ	5.20(4.80,5.40)	5.70(5.30,6.65) *	5.90(5.30,6.35) *	<0.001
Haemoglobin A1c (%)φ	5.45(5.30,5.70)	5.90(5.60,6.35) *	5.90(5.70,6.15) *	<0.001
Triglycerides (mmol/l)φ	0.83(0.60,1.22)	1.40(1.03,1.95) *	1.29(0.91,1.66) *	<0.001
TC (mmol/l)	4.91±0.85	5.35±0.99	5.34±0.90	0.064
ALT (U/L)φ	18.00(14.50,21.00)	26.00(19.00,34.50) *	21.00(19.00,35.00) *	<0.001
AST (U/L)φ	19.00(16.00,22.50)	22.00(19.00,26.00) *	21.00(18.00,26.00) *	0.026
Creatinine (μmol/l)	58.28±10.69	63.50±14.40	59.77±13.10	0.093

Data indicate means ± SD or medians (interquartile range). One-way ANOVAs with Fisher's *LSD post hoc* test were used to compare the differences among the three groups [*], $P < 0.05$ compared to lean; [#], $P < 0.05$ for difference between the obese versus overweight groups. Abbreviations: BMI, body mass index; FPG, fasting plasma glucose; AST, aspartate transaminase; ALT, alanine aminotransferase; TC, total cholesterol;
[φ]Log transformed before analysis.

Supplementary Table 3. Serum EMC10 concentrations at baseline and after 12 months of either bariatric surgery (n=50) or a combined hypocaloric diet and exercise program (n=50) in a white weight-loss cohort. Anthropometric and metabolic characteristics of study participants.

	Bariatric surgery (n=50)			Exercise/diet (n=50)		
	Baseline	12 months later	P-value*	Baseline	12 months later	*P*-value**
Sex, female/male (n)	30/20	30/20		36/14	36/14	
Age (years)	47.86±7.89	48.86±7.89		51.44±3.40	52.44±3.40	
BMI (kg/m²)	50.91±8.30	34.88±5.35	<0.001	35.14±4.47	33.42±5.58	0.001
FPG (mmol/l)	7.89±2.21	6.01±1.75	<0.001	7.85±2.38	6.13±1.45	<0.001
FPI (pmol/l)	140.50(90.20,251.00)	43.65(25.40,85.10)	<0.001	140.15(85.20,207.50)	93.15(49.20,155.20)	<0.001
HOMA-IR	7.15(3.98,10.38)	1.38(0.84,3.99)	<0.001	6.78(3.88,12.62)	3.43(1.72,6.32)	<0.001
HbA1c (%)	7.18±1.19	5.88±1.11	<0.001	6.98±0.79	6.35±0.78	<0.001
TC (mmol/l)	4.97 ± 1.17	4.70 ± 0.88	0.111	5.18 ± 1.10	5.18 ± 1.10	0.858
HDL-c (mmol/l)	1.29 ± 0.32	1.51 ± 0.43	<0.001	1.23 ± 0.23	1.35 ± 0.25	<0.001
LDL-c (mmol/l)	2.99 ± 0.94	2.76 ± 0.80	0.132	3.27 ± 0.93	3.26 ± 0.94	0.896
Triglycerides (mmol/l)	1.52(1.28,2.06)	1.20(0.99,1.54)	<0.001	1.71(1.37,2.32)	1.61(1.27,2.11)	0.005
FFA (mmol/l)	0.52±0.27	0.38±0.22	<0.001	0.56±0.28	0.48±0.28	<0.001
hsCRP (mg/dl)	4.96(2.27,7.23)	2.63(0.95,4.75)	0.003	4.31(2.89,8.26)	2.67(1.64,5.03)	0.021

Data indicate means ± SD or medians (interquartile range). Student's paired *t* test was used for before and after comparison. *P*-value* indicates the comparison of baseline and 12 months after bariatric surgery; *P*- value** indicates the difference before and after diet and exercise intervention. Abbreviations: BMI, body mass index; FPG, fasting plasma glucose; FPI, fasting plasma insulin; HbA1c,Haemoglobin A1c ; TC, total cholesterol; HDL-c, high density lipoprotein cholesterol; LDL-c, low density lipoprotein cholesterol; FFA, free fatty acid; hsCRP, high sensitivity C-reactive protein.

Supplementary Table 4: Primers used in the study

	Forward	Reverse
mMcp1	CCACTCACCTGCTGCTACTCAT	TGGTGATCCTCTTGTAGCTCTCC
mTnfa	GCCTCTTCTCATTCCTGCTTGT	GGCCATTTGGGAACTTCTCAT
mF4/80	TTTCCTCGCCTGCTTCTTC	CCCCGTCTCTGTATTCAAC
mscEmc10	TACTGGCACCTCATCCTGGG	TTAGGCCTCCGTTGGCGCTG
mAtgl	TAGCTAACAGTTGGGCTTCAC	CAGAGAGAACAGAGCAGCTTAC
mHsl	ACGGATACCGTAGTTTGGTGC	TCCAGAAGTGCACATCCAGGT
mAdrb3	GCTCTGTGTCTCTGGTTAGTTT	GTCCAAGATGGTGCTTAGAGAG
mPgc1a	GACAATCCCGAAGACACTACAG	AGAGAGGAGAGAGAGAGAGAGA
mUcp1	CTGCCAGGACAGTACCCAAG	TCAGCTGTTCAAAGCACACA
mTfam	GTCCATAGGCACCGTATTGCG	CCCATGCTGGAAAAACACTTCG
mGlut4	ACATACCTGACAGGGCAAGG	CGCCCTTAGTTGGTCAGAAG
mFasn	GCTGCGGAAACTTCAGGAAAT	AGAGACGTGTCACTCCTGGACTT
mSrebp1c	CCTCTGATCTCATGGCTCATAAC	CTAGGGAACTGTGTGTGTTTCT
mDio2	AAGGCTGCCGAATGTCAACGAATG	TGCTGGTTCAGACTCACCTTGGAA
mCox8b	GAACCATGAAGCCAACGACT	GCGAAGTTCACAGTGGTTCC
mElvol3	TCCGCGTTCTCATGTAGGTCT	GGACCTGATGCAACCCTATGA
mTBP	ACCCTTCACCAATGACTCCTATG	ATGATGACTGCAGCAAATCGC
scEMC10	TACTGGCACCTCATCCTGGG	TTAGGCCTCCGTTGGCGCTG
mEMC10	CTGTGGTTTGCCTGGTTTATG	CCTTGCTCACAGCAACTTTAAT
hscEMC10	ATACTGGCACATCATCCTGGGG	ACATCCATCAGGCCTCCTGTG
