# Supplementary material for: Secreted EMC10 is upregulated in human obesity and its neutralizing antibody prevents diet-induced obesity in mice
Source: Nat Commun. 2022 Nov 28;13:7323. doi: 10.1038/s41467-022-34259-9 (PMC9705309; doi:10.1038/s41467-022-34259-9)
Supplement: Supplementary file 3 — Reporting Summary [file 41467_2022_34259_MOESM3_ESM.pdf]

nature portfolio

Corresponding author(s): Chong Wee Liew

Last updated by author(s): Sep 21, 2022

Reporting Summary

Nature Portfolio wishes to improve the reproducibility of the work that we publish. This form provides structure for consistency and transparency in reporting. For further information on Nature Portfolio policies, see our Editorial Policies and the Editorial Policy Checklist.

Statistics

For all statistical analyses, confirm that the following items are present in the figure legend, table legend, main text, or Methods section.

n/a	Confirmed	
☐	☒	The exact sample size (*n*) for each experimental group/condition, given as a discrete number and unit of measurement
☐	☒	A statement on whether measurements were taken from distinct samples or whether the same sample was measured repeatedly
☐	☒	The statistical test(s) used AND whether they are one- or two-sided<br>*Only common tests should be described solely by name; describe more complex techniques in the Methods section.*
☐	☒	A description of all covariates tested
☐	☒	A description of any assumptions or corrections, such as tests of normality and adjustment for multiple comparisons
☐	☒	A full description of the statistical parameters including central tendency (e.g. means) or other basic estimates (e.g. regression coefficient) AND variation (e.g. standard deviation) or associated estimates of uncertainty (e.g. confidence intervals)
☐	☒	For null hypothesis testing, the test statistic (e.g. *F*, *t*, *r*) with confidence intervals, effect sizes, degrees of freedom and *P* value noted<br>*Give P values as exact values whenever suitable.*
☐	☒	For Bayesian analysis, information on the choice of priors and Markov chain Monte Carlo settings
☐	☒	For hierarchical and complex designs, identification of the appropriate level for tests and full reporting of outcomes
☐	☒	Estimates of effect sizes (e.g. Cohen's *d*, Pearson's *r*), indicating how they were calculated

Our web collection on statistics for biologists contains articles on many of the points above.

Software and code

Policy information about availability of computer code

Data collection	no software was used
Data analysis	Statistical analyses were performed under Statistical Package for Social Sciences version 22.0 (SPSS, Chicago, IL, USA) for human serum data. Imaje J (Version 1.0) for western blot analysis. GraphPad Prism version 7 & 9 and Statistical Package for Social Sciences version 20.0 for Statistical analysis of all other data.

For manuscripts utilizing custom algorithms or software that are central to the research but not yet described in published literature, software must be made available to editors and reviewers. We strongly encourage code deposition in a community repository (e.g. GitHub). See the Nature Portfolio guidelines for submitting code & software for further information.

Data

Policy information about availability of data

All manuscripts must include a data availability statement. This statement should provide the following information, where applicable:

- Accession codes, unique identifiers, or web links for publicly available datasets
- A description of any restrictions on data availability
- For clinical datasets or third party data, please ensure that the statement adheres to our policy

The data supporting the findings from this study are available within the manuscript and its supplementary information. Emc10 knockout mouse model genomic targeting DNA sequence (ENSMUSG00000008140). Source data are provided with this paper.

Human research participants

Policy information about [studies involving human research participants and Sex and Gender in Research.]

Reporting on sex and gender	Human research participants include both sexes who are randomly selected.
Population characteristics	For cross-sectional study, 240 white individuals which were either lean (BMI < 25 kg/m², n=30,average age 61.53, females =18), overweight (BMI 25-30 kg/m², n=22, average age 65.08, females =12) or patients with obesity (BMI > 30 kg/m², n=188, average age 47.93, females = 134) and 186 Chinese subjects which were either lean (BMI < 24 kg/m², n=32,average age 50.41, females =26), overweight (BMI 24-28 kg/m², n=115, average age 51.25, females =58) or individuals with obesity (BMI > 28 kg/m², n=39,average age 52.64, females =27) were included.<br>For follow-up studies, 100 white individuals with overweight or obesity (bariatric surgery, n=50,average age 47.86, female=30; hypocaloric diet and exercise, n=50, average age 51.44, female=36) were included.
Recruitment	For the purpose of our studies, we selected 240 white individuals from the Leipzig Obesity Biobank for whom serum and adipose tissues were available , and 100 white individuals underwent exercise and calorie restricted diet or bariatric surgery for whom serum were available at baseline and 12 months follow-up. To avoid selection and confounding bias, Chinese participants with matched age from Shanghai diabetes screening program were selected.
Ethics oversight	All studies were approved by the ethics committee of the University of Leipzig (approval numbers: 159-12-21052012 and 017-12-23012012) or human research ethics committee of Huashan hospital, following the principles of the Declaration of Helsinki. All subjects gave written informed consent before taking part in the study.

Note that full information on the approval of the study protocol must also be provided in the manuscript.

Field-specific reporting

Please select the one below that is the best fit for your research. If you are not sure, read the appropriate sections before making your selection.

☒ Life sciences ☐ Behavioural & social sciences ☐ Ecological, evolutionary & environmental sciences

For a reference copy of the document with all sections, see nature.com/documents/nr-reporting-summary-flat.pdf

Life sciences study design

All studies must disclose on these points even when the disclosure is negative.

Sample size	For cross-sectional study, 240 white individuals including 30 lean, 22 overweight and 188 with obesity were recruited. Serum EMC10 levels were missing in 3 lean individuals, 2 overweight individuals and 28 individuals with obesity. Finally, we included 207 individuals into our cross-sectional study of EMC10 serum concentrations. In a one-way ANOVA study, sample sizes of 27, 20, and 160 are obtained from the 3 groups whose means are to be compared. The total sample of 207 subjects achieves >99.99% power to detect differences among the means versus the alternative of equal means using an F test with a 0.05 significance level. The minimal required sample size in each group was assessed according to the difference EMC10 levels between the lean and obesity groups in white individuals. A study with 60 subjects will provide at least 90% power to detect an significant difference among 3 group assuming a type I error of 5% . The power and sample size analyses were calculated using PASS.11 software; In follow-up studies, we measured circulating Emc10 before and 12 months after a combined exercise and calorie restricted diet study (n=50 achieves 89% power with a significance level of 0.05 using a two-sided paired t-test), before and 12 months after bariatric surgery (n=50 achieves >99.99% power with a significance level of 0.05 using a two-sided paired t-test); 238 donors of paired omental and SC adipose tissue samples were used to measure adipose tissue Emc10 mRNA expression, who underwent abdominal surgery for cholecystectomy, weight reduction surgery, abdominal injuries or explorative laparotomy.
Data exclusions	We defined the following exclusion criteria: 1) Thyroid dysfunction, 2) alcohol or drug abuse, 3) pregnancy, 4) treatment with thiazolidinediones
Replication	The CLIA system had an intra- and inter-assay coefficient of variation at 3.3-13.8% and 12-16.3%, respectively
Randomization	Patients in the experimental and control groups were divided into subgroups based on their gender and BMI.
Blinding	Investigators were blinded to group allocation during data collection and data analysis

Reporting for specific materials, systems and methods

We require information from authors about some types of materials, experimental systems and methods used in many studies. Here, indicate whether each material, system or method listed is relevant to your study. If you are not sure if a list item applies to your research, read the appropriate section before selecting a response.

Materials & experimental systems

n/a	Involved in the study
☐	☒ Antibodies
☐	☒ Eukaryotic cell lines
☒	☐ Palaeontology and archaeology
☐	☒ Animals and other organisms
☒	☐ Clinical data
☒	☐ Dual use research of concern

Methods

n/a	Involved in the study
☒	☐ ChIP-seq
☒	☐ Flow cytometry
☒	☐ MRI-based neuroimaging

Antibodies

Antibodies used	Rabbit polyclonal antibodies to EMC10 (Phrenzer Biotechnology, Shanghai, China, 1:1000)<br>Mouse monoclonal antibodies (1F12, 4B12-1, 4B12-2, 4C2, 6B9, 1F12, 4B12-1, 1H11, 9:3D12) to EMC10 (Phrenzer Biotechnology, Shanghai, China). in vitro scEMC10 neutralization assay (1mg/ml); in vivo scEMC10 overexpression neutralization (9mg/kg BW); in vivo endogenous scEMC10 neutralization (3mg/kg BW).<br>anti-pS133-CREB (#9198; 1:1000; Cell Signaling Technology)<br>anti-total CREB (#9197; 1:1000; Cell Signaling Technology)<br>anti-p-P38MAPK (#4511; 1:1000; Cell Signaling Technology)<br>anti-total P38MAPK (#8690; 1:1000; Cell Signaling Technology)<br>anti-beta-actin (#66009; 1:1000; Proteintech)<br>anti-myc (#2278; 1:1000; Cell Signaling Technology)<br>anti-αtubulin (#66031; 1:5000; Proteintech)<br>anti-CREB1 (#A10826; 1:1000; ABclonal)<br>anti-Flag (1:3000; F7425; Sigma Aldrich)<br>anti-HA (1:3000; SC-7392; Santa Cruz Biotechnology)<br>anti-PKA C-α (1:1000; #5842; D38C6; Cell Signaling Technology).
Validation	https://www.cellsignal.com/products/primary-antibodies/phospho-creb-ser133-87g3-rabbit-mab/9198<br>https://www.cellsignal.com/products/primary-antibodies/creb-48h2-rabbit-mab/9197<br>https://www.cellsignal.com/products/primary-antibodies/phospho-p38-mapk-thr180-tyr182-d3f9-xp-rabbit-mab/4511<br>https://www.cellsignal.com/products/primary-antibodies/p38-mapk-d13e1-xp-rabbit-mab/8690<br>https://www.ptglab.com/products/Pan-Actin-Antibody-66009-1-Ig.htm<br>https://www.cellsignal.com/products/primary-antibodies/myc-tag-71d10-rabbit-mab/2278<br>https://www.ptglab.com/products/tubulin-Alpha-Antibody-66031-1-Ig.htm<br>https://abclonal.com/catalog-antibodies/CREB1MonoclonalAntibody/A10826<br>https://www.sigmaaldrich.com/US/en/product/sigma/f7425<br>https://www.scbt.com/p/ha-probe-antibody-f-7<br>https://www.cellsignal.com/products/primary-antibodies/pka-c-a-d38c6-rabbit-mab/5842<br>Rabbit polyclonal antibodies to Emc10 and mouse monoclonal antibodies to EMC10 information were under submission for patent.

Eukaryotic cell lines

Policy information about cell lines and Sex and Gender in Research

Cell line source(s)	Hela cells and HEK293t cells were purchased from ATCC
Authentication	Short Tandem Repeat (STR) DNA profiling were used to authenticate cell line
Mycoplasma contamination	All cell lines tested negative for mycoplasma contamination
Commonly misidentified lines<br>(See ICLAC register)	There were no commonly misidentified cell lines used in the study

Animals and other research organisms

Policy information about studies involving animals; ARRIVE guidelines recommended for reporting animal research, and Sex and Gender in Research

Laboratory animals	Male mice on C57BL/6 background (6-10 weeks) were used in this study. C57BL/6J and ob/ob male mice were obtained from the Jackson Laboratory (USA). Exact age information for each experiment can be found in method. Housing condition: Temperature: 20-24 degree C; humidity: 45-65%.
Wild animals	The study didn't involve wild animals
Reporting on sex	All animals included in this study are male.
Field-collected samples	the study did not involve samples collected from field

Ethics oversight	The animal protocols (#15-026 &18-010) were approved by the Institutional Animal Care and Use Committee (IACUC) of University of Illinois at Chicago.

Note that full information on the approval of the study protocol must also be provided in the manuscript.
